# Online Application of Imagery Rescripting for Pathological Affective Dependence (ImRs-PAD) in Survivors of Intimate Partner Violence: Results From a Multiple Case Series

**DOI:** 10.32872/cpe.22027

**Published:** 2026-08-31

**Authors:** Erica Pugliese, Allison Uvelli, Arnold A. P. van Emmerik, Arnoud Arntz

**Affiliations:** 1 Clinical Psychology Group, Department of Psychology, University of Amsterdam, Amsterdam, The Netherlands; 2 School of Cognitive Psychology, Rome, Italy; 3 Department of Medical Science, Surgery and Neurosciences, University of Siena, Siena, Italy; Philipps-University of Marburg, Marburg, Germany

**Keywords:** intimate partner violence (IPV), pathological affective dependence (PAD), relational trauma, imagery rescripting (ImRs), mechanisms of change in ImRs, psychotherapy

## Abstract

**Background:**

This study examined the feasibility and preliminary clinical indications of an imagery rescripting intervention integrating the Pathological Affective Dependence framework (ImRs-PAD) in survivors of Intimate Partner Violence (IPV). Pathological Affective Dependence (PAD) refers to a trauma-related relational vulnerability associated with difficulties in disengaging from abusive intimate relationships.

**Method:**

A multiple case series design was used with three female IPV survivors referred by a mental health center in Italy (mean age = 31.3 years, *SD* = 2.08). All participants reported histories of abusive relationships, marked difficulties with separation, and clinically significant levels of PAD. Participants received an online ImRs-PAD intervention. Repeated self-report measures assessed changes in PAD, trauma-related symptoms, depression, anxiety, somatic symptoms, resilience, self-compassion, and psychological well-being.

**Results:**

Across cases, reductions were observed in PAD, depressive and anxiety symptoms, and trauma-related and somatic complaints, alongside increases in resilience, self-compassion, and overall psychological well-being. The online delivery of the intervention was feasible, with no technical or safety issues reported.

**Conclusion:**

The findings provide preliminary support for the potential clinical relevance of ImRs-PAD for IPV survivors. Addressing PAD may facilitate disengagement from violent partners and reduce the risk of re-exposure to severe forms of violence.

## Highlights

- Online imagery rescripting was feasible and safe for IPV survivors with pathological affective dependence.
- The intervention was associated with reductions in pathological affective dependence and trauma-related symptoms.
- Improvements were observed in self-compassion, resilience, and psychological well-being.
- Addressing unmet relational needs may support disengagement from abusive relationships.

Intimate Partner Violence (IPV) represents a pervasive public health issue with profound psychological, physical, and social consequences, particularly for women (Jewkes & Abrahams, 2026). The World Health Organization (WHO, 2024) defines IPV as harmful behaviors by a current or former intimate partner, including physical aggression, sexual coercion, psychological abuse, and controlling behaviors. Remaining in abusive relationships is influenced by structural and individual obstacles, including economic, social, and cultural pressures, as well as the perpetrator’s psychological characteristics (i.e., anger problems, anxiety, depression, suicidal behaviour, personality disorders, alcoholism, or problem gambling; Cuesta-García & Crespo, 2022; Neal & Edwards, 2017). Over time, increasing attention has also been directed toward the relational dynamics between victims and perpetrators of IPV (Pugliese et al., 2024). Within this context, Pathological Affective Dependence (PAD) has been proposed as an emerging clinical construct describing a vulnerability that may increase the likelihood of remaining in or returning to abusive relationships (Castillo-Gonzáles et al., 2024; Pugliese et al., 2023). PAD is a relational condition characterized by a persistent and intense need to maintain a specific intimate relationship despite its dysfunctional nature, including the presence of psychological distress or interpersonal violence (Pugliese et al., 2023). Individuals with PAD remain trapped in these dangerous relationships as they experience an internal conflict: the compulsion to save the relationship, even at the expense of their own well-being, and the desire to protect themselves from further harm. Two conditions typically fuel this internal struggle: partner abuse and perceived inability to leave the relationship. PAD encompasses both conditions and the resulting internal struggle (Pugliese, Uvelli, et al., 2025).

PAD can be conceptualized as both a trait—reflecting enduring dysfunctional relational patterns—and a state, referring to the situational activation of these patterns within a specific relationship, particularly in abusive or violent contexts (Pugliese, Uvelli, et al., 2025). In addition, PAD appears to be associated with reduced cognitive and metacognitive functioning in IPV survivors, which may partly explain difficulties in help-seeking and relational disengagement (Pugliese, Papa, et al., 2025). According to the PAD theory (Pugliese et al., 2023), the etiology of PAD lies in the chronic frustration of at least one of three core relational needs—love (acceptance and care), dignity (being respected and valued), and safety (physical and emotional security)—within early caregiving relationships (Papa & Pugliese, 2025; Papa et al., 2026; Smith‐Marek et al., 2015; Speranza et al., 2022). In this framework, relational trauma is conceptualized as the cumulative impact of persistent unmet relational needs. In adulthood, these experiences may consolidate into trait PAD, understood as a stable pattern of dysfunctional relational functioning. Individuals with such histories often engage in intimate relationships with an implicit reparative aim, seeking to fulfill unmet developmental needs (Pezzoli et al., 2025; Phillips et al., 2025). However, these efforts are frequently directed toward relational contexts that resemble early caregiving environments, thereby reactivating and reinforcing maladaptive interpersonal patterns (Pugliese et al., 2023). Within this process, current relationships may elicit state PAD, defined as the situational manifestation of these dynamics within a specific relational context. This condition is typically characterized by ongoing abuse, a perceived inability to disengage, and a persistent conflict between separation and relationship maintenance. Such dynamics contribute to both the maintenance of abusive relationships and the perpetuation of maladaptive relational cycles. From this perspective, PAD may be understood as the enduring relational imprint of repeated relational trauma, predisposing individuals to recurrent patterns of dysfunction and abuse (Silvestri et al., 2025; Speranza et al., 2022). Despite its clinical significance, there remains a notable lack of interventions specifically designed to address psychological mechanisms rooted in early trauma that increase vulnerability to revictimization—mechanisms that are central to PAD.

## Study Rationale

Imagery Rescripting (ImRs) is one promising evidence-based intervention to address the unmet needs central to PAD. This experiential technique, originally developed within Schema Therapy (Arntz & Weertman, 1999), allows clients to revisit early aversive memories, fantasies, or nightmares and transform their emotional meaning (Müller-Engelmann & Steil, 2017; Nilsson et al., 2019). One of the hypothesized mechanisms of change in ImRs is the fulfillment of unmet needs (Koetsier et al., 2024). To our knowledge, however, no study has examined whether satisfying unmet needs from childhood and adulthood using guided imagery may be beneficial for survivors of IPV presenting with PAD (for a review, see Kip et al., 2023; Kroener et al., 2023). Specifically, it remains unclear whether, after these needs are addressed in imagination, survivors report reductions in PAD. Additionally, previous research on treatments for women IPV survivors has largely focused on PTSD, depression, anxiety, and self-esteem (Emirza & Uzun, 2024), often overlooking complex PTSD (ICD-11, 2018) symptoms—namely disturbances in self-organization, including affective dysregulation, negative self-concept, and relational difficulties—linked to ongoing trauma (Cloitre et al., 2018), as well as positive outcomes such as resilience and self-compassion. ImRs-PAD is expected to foster a more supportive and protective stance toward the vulnerable self, potentially increasing self-compassion. This is particularly relevant in survivors of IPV, who often experience high levels of self-criticism for not leaving the relationship earlier, despite recognizing its dysfunctional or abusive nature (Crapolicchio et al., 2021). Such self-criticism may be further reinforced by self-blame processes, as perpetrators frequently attribute responsibility for the violence to the victim (Naismith et al., 2024). This is consistent with the PAD construct, particularly the internal conflict dimension, which reflects the struggle between recognizing that the relationship is harmful and feeling unable to separate from the partner (Pugliese, Uvelli, et al., 2025). Moreover, despite substantial therapeutic and support efforts, many IPV survivors remain resistant to change within existing care systems (Paphitis et al., 2022). Existing evidence-based interventions for IPV survivors primarily target symptom reduction (e.g., PTSD, depression, anxiety) and may not directly address the underlying relational mechanisms that maintain dependence on abusive partners. These approaches may be less effective in modifying unmet relational needs and the associated internal conflict that characterizes PAD. The present study aims to address this gap by framing PAD as a relational pathway through which traumatic family and partner experiences may contribute to persistent difficulties in disengaging from abusive relationships. Addressing PAD may therefore represent a trauma-informed pathway to reduce re-exposure to violence, complementing symptom-focused PTSD treatments.

## The Current Study

To address this gap, the present study explores the feasibility and preliminary effectiveness of an online imagery rescripting intervention (ImRs-PAD) tailored for female survivors of IPV with clinically meaningful PAD symptoms. Using an exploratory multiple case series design, the intervention specifically targets three core unmet needs—love, dignity, and safety—identified from participants’ autobiographical narratives and relational histories. In addition to evaluating the feasibility of online delivery, the study examines whether ImRs-PAD reduces PAD, complex post-traumatic stress, depression, anxiety, and somatic complaints while enhancing psychological well-being, self-compassion, and resilience. Online access is particularly relevant for IPV survivors who often face barriers to in-person care due to ongoing partner control or surveillance (van Gelder et al., 2023). The present study is an independent online multiple case series conducted in Italy, designed to provide a preliminary evaluation of the protocol prior to the implementation of a preregistered in-person multiple baseline study trial (ClinicalTrials.gov Identifier: NCT06670326). The preregistered trial is conducted in collaboration with several anti-violence centers and is currently ongoing in the Netherlands.

## Hypotheses

We formulated a set of hypotheses organized into primary and secondary outcomes, designed to capture the direct effects of the intervention at different stages of the protocol.

- *Primary Outcomes:* Participants will show significant reductions in both state PAD and trait PAD from pre-treatment to post-treatment, with effects maintained at follow-up.
- *Secondary Outcomes:* Participants will show significant increases in resilience, self-compassion, and psychological well-being, as well as reductions in depression, anxiety, and complex post-traumatic stress symptoms from pre-treatment to post-treatment, with improvements sustained or enhanced at follow-up.

## Method

### Participants

Three female participants were referred by a psychiatrist from a mental health center in Italy to participate in the current study. All presented with a history of abusive relationships with current or former partners and reported marked difficulties in achieving or maintaining separation despite recognizing the need to do so. The mean age was 31.3 years (*SD* = 2.08). All participants were unmarried at the time of treatment and reported recurrent difficulties in maintaining stable and autonomous romantic relationships. They also showed a marked inability to leave dysfunctional relationships and experienced exacerbations of depressive and anxiety symptoms during separation phases. All patients referred during the study period who met the inclusion criteria and provided informed consent were included. Inclusion criteria were sufficient fluency in Italian to complete the study procedures, age between 18 and 60 years, and clinically meaningful levels of PAD, as indicated by a score of 47 or higher on the Pathological Affective Dependence Scale – State and Trait versions (PADS-S, PADS-T; Pugliese, Uvelli, et al., 2025). Exclusion criteria were assessed through a comprehensive clinical interview by a licensed psychologist and psychotherapist with 10 years of clinical experience. These included the evaluation of current and past psychiatric conditions, substance use, medical history, and ongoing treatments. The assessment aimed to rule out the presence of psychotic disorders, severe substance use disorder, intellectual disability, debilitating chronic medical conditions, prior receipt of imagery rescripting–based therapy, and current psychopharmacological treatment. Detailed descriptions of individual cases included in this multiple case series are reported in Appendix A.

### Procedures

The intervention was conducted by two licensed clinical psychologists, both formally trained in cognitive-behavioral therapy (CBT) for PAD, and engaged in peer supervision throughout the treatment process. One therapist, with over 4 years of experience in both clinical practice and research, delivered the treatment to all three participants. The second therapist, with over 10 years of experience in both clinical practice and research, provided clinical supervision and administered the assessment measures. Three clients participated in a 5-month program consisting of weekly individual sessions (approximately 20 sessions each, including 4–6 assessment sessions, 13 treatment sessions, and the remaining sessions focused on follow-up).

All treatment sessions were conducted online via Google Meet using secure, individualized access links. For each session, a unique private link was generated and sent to the participant’s personal email address the day before the appointment. Access to the session was restricted to the invited participant, ensuring confidentiality and controlled access. A different link was used for each session and for each participant.

Before the intervention, all clients underwent a comprehensive psychological assessment. All self-report measures were completed independently by participants outside the therapy sessions. Therapists did not use questionnaire scores to guide the intervention and had no access to interim results during treatment. The treatment program started in March 2024 and concluded in August 2025. All participants provided informed consent, and the study was approved by the Ethics Committee of the Scuola di Psicoterapia Cognitiva (protocol number N. Pr 2/24).

The treatment component, ImRs-PAD, is a trauma-informed intervention based on Imagery Rescripting (ImRs), consisting of 13 weekly individual sessions (50 minutes each), including one psychoeducation session followed by 12 ImRs sessions. Grounded in PAD theory (Pugliese et al., 2023), ImRs-PAD aims to address chronic frustration of core relational needs for love, dignity, and safety across early family experiences, abusive intimate relationships, and anticipated future relational challenges. Across stages, responsibility for rescripting progressively shifts from the therapist to the client, fostering autonomy, affect regulation, and relational agency. ImRs-PAD is organized into three stages, each specifically targeting the reduction of PAD traits, PAD state, and relapse.

### Measures

The Pathological Affective Dependence Scale (PADS; Pugliese, Uvelli, et al., 2025) is a 17-item self-report questionnaire available in two parallel forms that measure state (PADS-S) and trait (PADS-T) affective dependence, respectively. It assesses three dimensions of pathological dependence, including internal conflict (IC), inability to separate from the partner (IS), and partner abuse (PA). Items are rated on a 5-point Likert scale (1 = *not at all*, 5 = *always*). The total score ranges from 17 to 85, with higher scores indicating greater levels of affective dependence. In the original validation study (Pugliese, Uvelli, et al., 2025), Cronbach’s α was .89 for both the state and trait versions. Example items include *“I am aware that I am suffering in this relationship, but at the same time, I cannot leave it”* (IC); *“I would do anything in order not to lose my importance in my partner’s sight”* (IS); and *“My partner devalues me”* (PA). In this study, the PADS was administered at baseline (T0), after every session (T1-T12), and at follow-up.

The Self-Compassion Scale (SCS; Neff, 2003) is a 26-item questionnaire designed to measure six dimensions of self-compassion, including self-kindness, self-judgment, common humanity, isolation, mindfulness, and over-identification. Each item is rated on a 5-point Likert scale (1 = *almost never*, 5 = *almost always*). The total score ranges from 26 to 130, with higher scores reflecting greater self-compassion. To obtain the scores for the subscales, the mean of the items belonging to each subscale should be calculated. Cronbach’s α in the original study was .92 for the total score and between .75 and .81 for the subscales. Example items include *“I try to be loving toward myself when I’m feeling emotional pain”* and *“I’m disapproving and judgmental about my own flaws and inadequacies.”* In this study, the SCS was administered at baseline (T0), after 6 sessions (T6), after 12 sessions (T12), and at follow-up.

The Patient Health Questionnaire (PHQ; Kroenke et al., 2001, 2002; Spitzer et al., 2006) is a widely used self-report instrument that screens for somatic, anxiety, and depressive symptoms. It consists of 31 items across different modules, each rated on a 4-point Likert scale (0 = *not at all*, 3 = *nearly every day*). The score range differs for each module (e.g., PHQ-9 depression: 0–27; PHQ-15 somatic symptoms: 0–45; GAD-7 anxiety: 0–21). Higher scores indicate greater symptom severity. Cronbach’s α values in validation studies ranged from .86 to .89 (Kroenke et al., 2001, 2002; Spitzer et al., 2006). Example items include *“Over the last 2 weeks, how often have you had little interest or pleasure in doing things?”* and *“Over the last 2 weeks, how often have you felt nervous, anxious, or on edge?”* In this study, the PHQ was administered at T0, T6, T12, and follow-up.

The Ego-Resiliency Scale – Revised (ER89-R; Alessandri et al., 2007) is a 10-item self-report questionnaire designed to assess psychological resilience, particularly the ability to adapt to changing circumstances flexibly. Items are rated on a 4-point Likert scale (1 = *does not apply at all*, 4 = *applies very strongly*). The total score ranges from 10 to 40, with higher scores indicating greater ego-resiliency. Cronbach’s α in the Italian validation was .76 (Alessandri et al., 2007). Example items include: *“I quickly get over and recover from being startled”* and *“I enjoy dealing with new and unusual situations.”* The ER89-R was administered at T0, T6, T12, and follow-up.

The International Trauma Questionnaire (ITQ; Cloitre et al., 2018) is an 18-item measure assessing symptoms of post-traumatic stress disorder (PTSD) and complex PTSD (CPTSD) according to ICD-11 criteria. Each item is scored on a 5-point Likert scale (0 = *not at all*, 4 = *extremely*). The total score ranges from 0 to 72, with higher scores indicating greater symptom severity. Cronbach’s α in the original validation study (Cloitre et al., 2018) ranged from .88 to .94 across the different subscales. Example items include: *“When I am reminded of the trauma, I get intense physical reactions (for example, sweating, heart beating fast, nausea)”* and *“I feel worthless because of the trauma.”* In this study, the ITQ was administered at T0, T6, T12, and follow-up.

The Psychological General Well-Being Index – Short (PGWB-S; Grossi et al., 2006) is a 6-item self-report scale measuring overall subjective well-being. Each item is rated on a 6-point Likert scale (0 = *none of the time*, 5 = *all of the time*), with total scores ranging from 0 to 30. Higher scores indicate better psychological well-being. Cronbach’s α in validation studies (Grossi et al., 2006) ranged from .85 to .90. Example items include *“Have you been bothered by nervousness or your ‘nerves’ during the past month?”* and *“How often have you felt cheerful and light-hearted?”* The PGWB-SF was administered at T0, T6, T12, and follow-up.

The Young Schema Questionnaire – Short Form (YSQ-SF; Young & Brown, 2005) is a 90-item self-report questionnaire designed to assess 18 early maladaptive schemas. Items are rated on a 6-point Likert scale (1 = *completely untrue of me*, 6 = *describes me perfectly*). The total score ranges from 90 to 540, with higher scores reflecting stronger schema endorsement. Cronbach’s α for the subscales in the original validation study (Young & Brown, 2005) ranged from .83 to .96. Example items include: *“I worry that people I feel close to will leave me or abandon me”* and *“I feel that people will take advantage of me.”* In this study, the YSQ-SF was administered only at baseline (T0).

All self-report measures were administered using standardized procedures to minimize response bias and social desirability effects. Questionnaires were completed independently by participants online via a secure survey platform. Measures were administered and collected by a research team member not involved in therapy, and therapists had no access to questionnaire responses at any assessment point, ensuring a clear separation between clinical and research roles.

### Statistical Analysis

Data analysis was conducted in two steps. First, pre- (T0) and post-treatment (T12) scores were compared for each case using the Reliable Change Index (RCI; Jacobson & Truax, 1991), which expresses the ratio between the observed change and the standard error of Italian measurement. Values greater than 1.96 were considered statistically significant, indicating a reliable change. The RCI was selected because it allows the evaluation of clinically meaningful changes at the individual level, which is particularly appropriate for case-series designs in small samples. Second, differences were compared using the RCI across intermediate and follow-up phases (T0–T6, T6–T12, T12–FU, and T0–FU), to capture both short-term and long-term changes. All analyses were performed at the individual level, in line with the descriptive and exploratory nature of case-series designs.

## Results

Across all three cases, results showed consistent improvements on every measure from baseline (T0) to follow-up (FU). Specifically, both the state and trait versions of the PADS revealed marked reductions, with final scores approaching the minimum possible value for each participant. In parallel, self-compassion (SCS) increased steadily, with Case 1 reaching the highest overall levels at follow-up. Depressive, somatic, and anxiety symptoms measured with the PHQ decreased substantially in all participants. At the same time, psychological well-being (PGWB) showed a progressive rise, particularly in Case 1 and Case 3, where scores nearly doubled or tripled relative to baseline. Ego-resiliency (ER-89-R) also increased across cases, reflecting greater adaptive functioning over time. Finally, trauma-related symptoms (ITQ) declined sharply, with Case 1 and Case 2 showing the fastest early reductions, while Case 3 improved more gradually but reached substantially lower symptom levels at follow-up. Overall, the data highlight a consistent pattern of symptom reduction and functional improvement across all domains, with convergence of outcomes among the three clients despite some differences in the pace of change (see Table 1).

**Table 1:** Pre-Treatment (T0), Half-Treatment (T6), Post-Treatment (T12), and Follow-Up (FU) Scores of Participants on All Outcome Measures

Variable	Case 1 T0-T6-T12-FU	Case 2 T0-T6-T12-FU	Case 3 T0-T6-T12-FU
PADS-S	69-30-18-17	71-64-39-20	68-33-22-17
PADS-T	75-38-30-18	72-66-39-22	61-32-22-17
SCS	1.53-2.86-4.08-4.50	2.30-2.50-3.05-3.80	1.66-2.23-2.46-2.86
PHQ	49-25-22-10	25-19-13-8	31-23-20-10
PGWB	47.58-91.5-106.14-109.8	43.92-47.58-62.22-75.13	14.64-25.62-30.22-41.14
ER-89-R	38-46-48-50	37-43-44-52	30-40-42-45
ITQ	59-16-11-6	51-34-18-10	59-41-33-20

*Note*. T0 = pre-treatment; T6 = half-treatment; T12 = post-treatment; FU = follow-up; PADS-S = Pathological Affective Dependence Scale-State version; PADS-T = Pathological Affective Dependence Scale-Trait version; SCS = Self-Compassion Scale; PHQ = Patient Health Questionnaire; PGWB = Psychological General Well-Being Index; ER-89-R = Ego-Resiliency Scale-Revised; ITQ = International Trauma Questionnaire.

All three participants demonstrated statistically reliable improvements across the outcome measures, with differences in timing and magnitude (Table 2). A concise overview of the temporal pattern of reliable changes across outcome measures is reported in Table 3.

**Table 2:** Reliable Change Index (RCI) of the Outcome Measures Over Time

Variable	Case 1 T0-T12 / T0-FU / T0-T6 / T6-12 / T12-FU	Case 2 T0-T12 / T0-FU / T0-T6 / T6-12 / T12-FU	Case 3 T0-T12 / T0-FU / T0-T6 / T6-12 / T12-FU
PADS-S	8.79* / 8.96* / 6.72* / 2.07* / 0.17	5.52* / 8.79* / 1.21 / 4.31* / 3.27*	7.93* / 8.79* / 0.69 / 1.90 / 0.86
PADS-T	7.66* / 9.70* / 6.30* / 1.36 / 2.04*	5.62* / 8.51* / 1.02 / 4.59* / 2.89*	6.64* / 7.49* / 4.93* / 1.70 / 0.85
SCS	-7.08* / -9.00* / -3.96* / -3.70* / -1.27	-2.08* / -4.55* / -0.56 / -1.67 / -2.27*	-2.22* / -3.64* / -1.58 / -0.70 / -1.21
PHQ	6.53* / 9.43* / 5.80* / 0.73 / 2.90*	2.90* / 4.11* / 1.45 / 1.45 / 1.21	2.66* / 5.08* / 1.93 / 0.73 / 2.42*
PGWB	-19.91* / -21.16* / -14.93* / -4.98* / -1.24	-6.22* / -10.61* / -1.24 / -4.98* / -4.39*	-5.30* / -9.01* / -3.73* / -1.56 / -3.71*
ER-89-R	-8.37* / -10.04* / -6.69* / -1.67 / -1.67	-5.86* / -12.55* / -5.02* / -0.84 / -6.69*	-10.04* / -12.55* / -8.37* / -1.67 / -2.51*
ITQ	11.87* / 13.11* / 10.63* / 1.24 / 1.24	8.16* / 10.14* / 4.20* / 3.96* / 1.98*	6.43* / 9.64* / 4.45* / 1.98* / 3.21*

*Note.* PADS-S = pathological affective dependence scale-state version; PADS-T = pathological affective dependence-trait version; SCS = self-compassion scale; PHQ = patient health questionnaire; PGWB = psychological general well-being index; ER-89-R = ego-resiliency-89-revised; ITQ = international trauma questionnaire. T0–T12 = comparison between pre-treatment and the last treatment session; T0–FU = comparison between pre-treatment and follow-up; T0–T6 = comparison between pre-treatment and mid-treatment (after the sixth imagery with rescripting session); T6–T12 = comparison between mid-treatment and the last treatment session; T12–FU = comparison between the last treatment session and follow-up.

*Significant results at |RCI| > 1.96.

**Table 3:** Summary of Temporal RCI Changes Across Assessment Phases

Variable	Case 1 T0-T12 / T0-T6 / T6-T12 / T12-FU / T0-FU	Case 2 T0-T12 / T0-T6 / T6-T12 / T12-FU / T0-FU	Case 3 T0-T12 / T0-T6 / T6-T12 / T12-FU / T0-FU
PADS-S	8.79* / 6.72* / 2.07* / 0.17 / 8.96*	5.52* / 1.21 / 4.31* / 3.27* / 8.79*	7.93* / 0.69 / 1.90 / 0.86 / 8.79*
PADS-T	7.66* / 6.30* / 1.36 / 2.04* / 9.70*	5.62* / 1.02 / 4.59* / 2.89* / 8.51*	6.64* / 4.93* / 1.70 / 0.85 / 7.49*
SCS	-7.08* / -3.96* / -3.70* / -1.27 / -9.00*	-2.08* / -0.56 / -1.67 / -2.27* / -4.55*	-2.22* / -1.58 / -0.70 / -1.21 / -3.64*
PHQ	6.53* / 5.80* / 0.73 / 2.90* / 9.43*	2.90* / 1.45 / 1.45 / 1.21 / 4.11*	2.66* / 1.93 / 0.73 / 2.42* / 5.08*
PGWB	-19.91* / -14.93* / -4.98* / -1.24 / -21.16*	-6.22* / -1.24 / -4.98* / -4.39* / -10.61*	-5.30* / -3.73* / -1.56 / -3.71* / -9.01*
ER-89-R	-8.37* / -6.69* / -1.67 / -1.67 / -10.04*	-5.86* / -5.02* / -0.84 / -6.69* / -12.55*	-10.04* / -8.37* / -1.67 / -2.51* / -12.55*
ITQ	11.87* / 10.63* / 1.24 / 1.24 / 13.11*	8.16* / 4.20* / 3.96* / 1.98* / 10.14*	6.43* / 4.45* / 1.98* / 3.21* / 9.64*

*Note*. RCI = reliable change index; PADS-S = Pathological Affective Dependence Scale-State version; PADS-T = Pathological Affective Dependence Scale-Trait version; SCS = Self-Compassion Scale; PHQ = Patient Health Questionnaire; PGWB = Psychological General Well-Being Index; ER-89-R = Ego-Resiliency Scale-Revised; ITQ = International Trauma Questionnaire.

*Significant results at |RCI| > 1.96.

Case 1 showed the most rapid and extensive response. Substantial improvements were evident already at mid-treatment, with reliable reductions in pathological affective dependence, general psychopathology, and trauma-related symptoms, as well as increases in well-being, self-compassion, and resilience. These gains were largely maintained and consolidated through post-treatment and follow-up. Case 2 exhibited a more gradual trajectory. Early changes were limited, but significant progress emerged between mid- and post-treatment, particularly in affective dependence, trauma symptoms, and psychological well-being. Additional gains were observed at follow-up, including further improvements in resilience. Case 3 also showed a slower but consistent pattern of change. Improvements were selective at mid-treatment, mainly in resilience and affective dependence, and became more evident by post-treatment. Further progress occurred at follow-up, especially in well-being and trauma-related symptoms. Taken together, the results indicate that while the three participants followed different trajectories, they all achieved clinically meaningful improvements by the end of treatment and maintained or enhanced these gains at follow-up.

**Figure 1:**
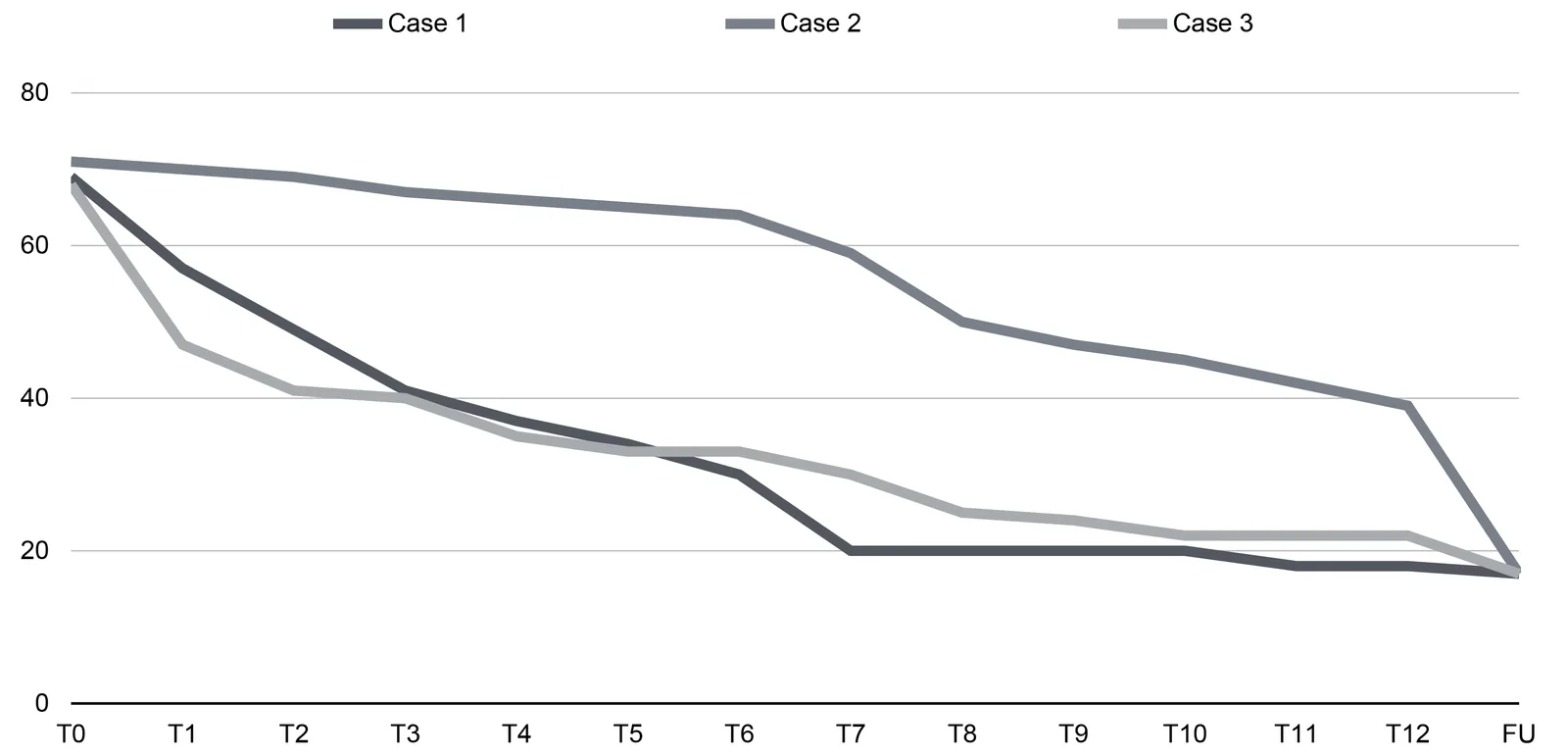
Time Series of State Pathological Affective Dependence: Changes in the Total Score of PADS-S *Note.* PADS-S = Pathological Affective Dependence Scale-State version; T0 = pre-treatment; T6 = half-treatment; T12 = post-treatment; FU = follow-up. Higher scores indicate higher state pathological affective dependence.

**Figure 2:**
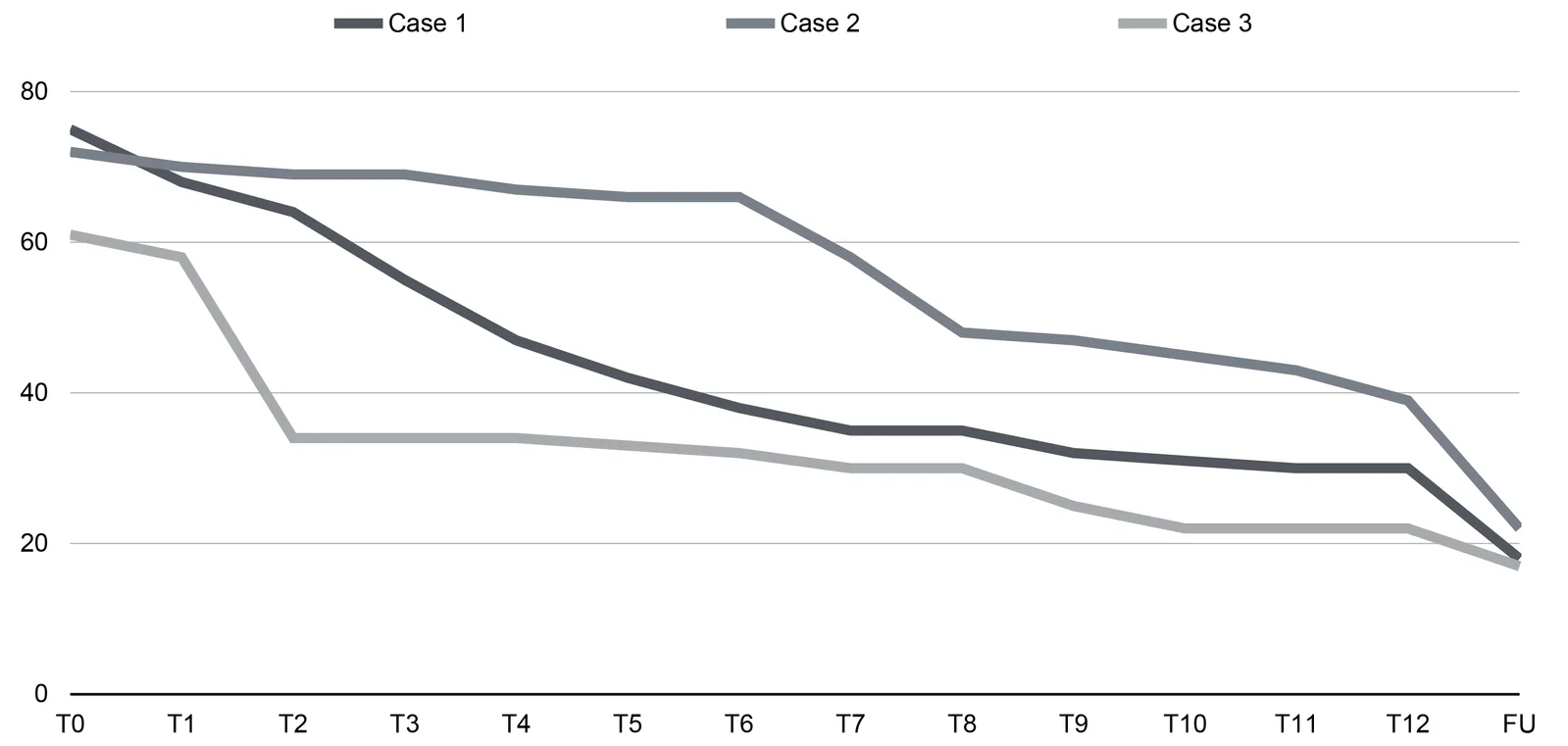
Time Series of Trait Pathological Affective Dependence: Changes in the Total Score of PADS-T *Note.* PADS-T = Pathological Affective Dependence Scale-Trait version; T0 = pre-treatment; T6 = half-treatment; T12 = post-treatment; FU = follow-up. Higher scores indicate higher trait pathological affective dependence.

**Figure 3:**
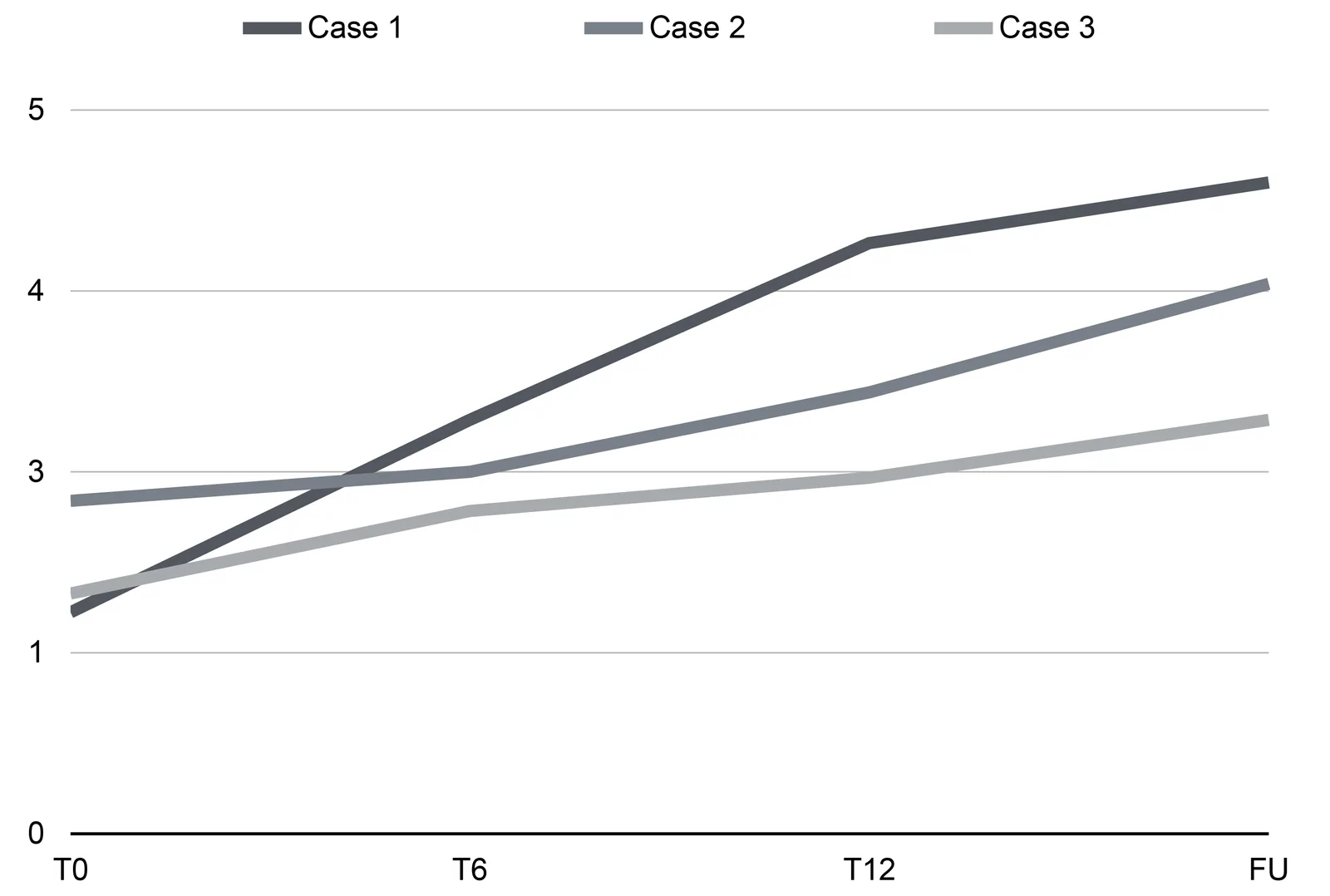
Self-Compassion Score Over Time: Changes in the Total Score of SCS *Note.* SCS = Self-Compassion Scale; T0 = pre-treatment; T6 = half-treatment; T12 = post-treatment; FU = follow-up. Higher scores indicate higher self-compassion.

**Figure 4:**
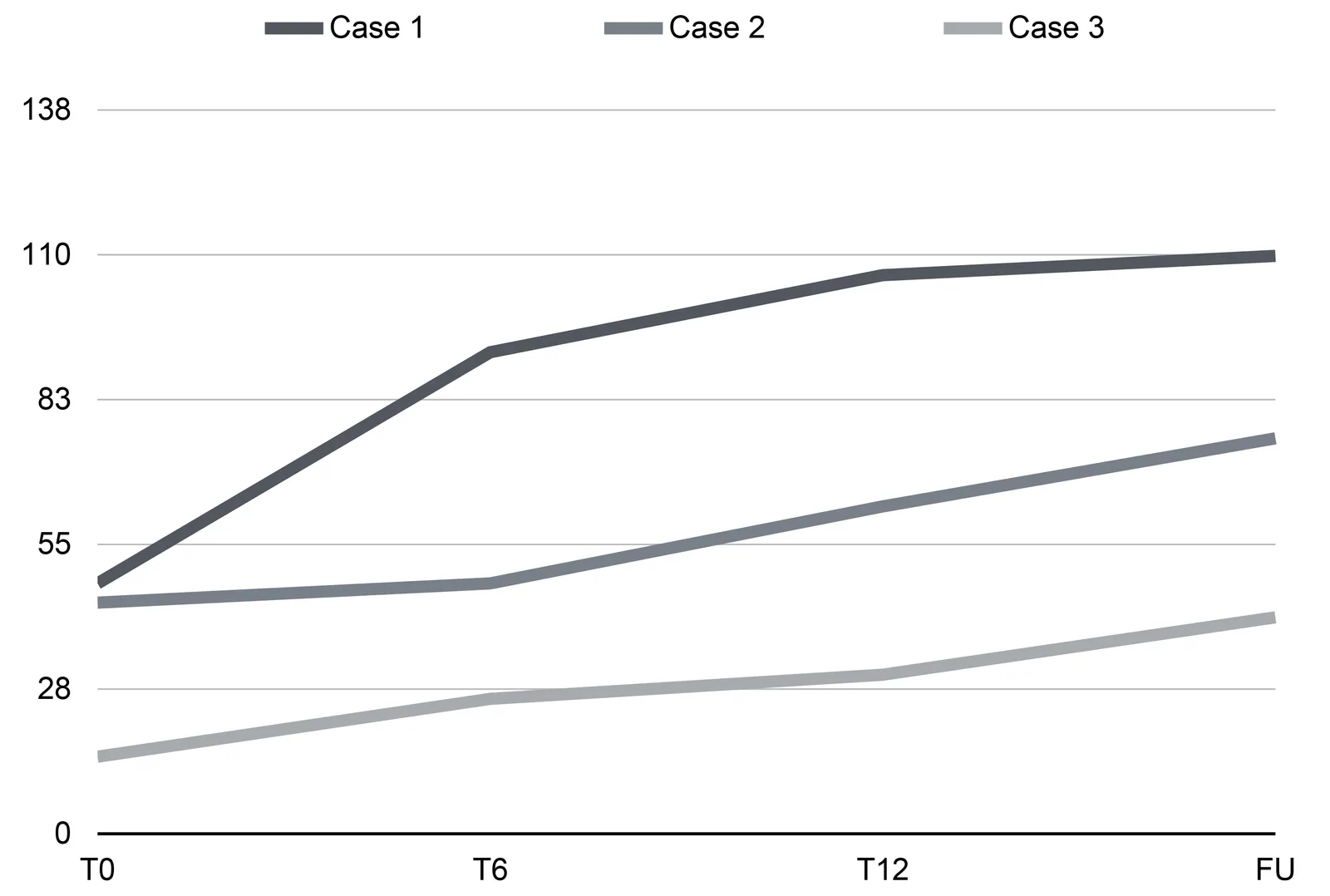
Psychological Well-Being Over Time: Changes in the Total Score of PGWB *Note.* PGWB = Psychological General Well-Being Index; T0 = pre-treatment; T6 = half-treatment; T12 = post-treatment; FU = follow-up. Higher scores indicate higher psychological well-being.

**Figure 5:**
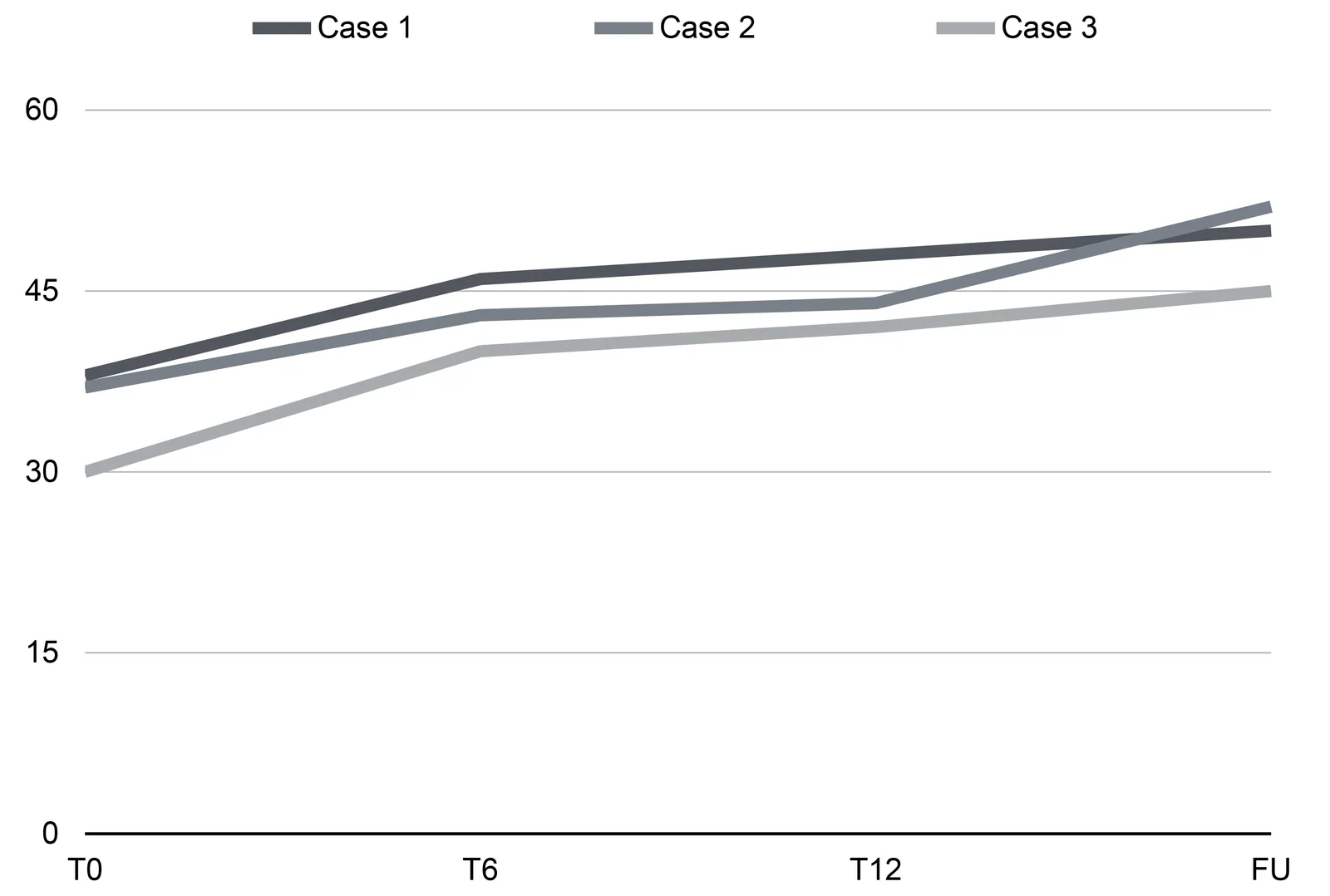
Resilience Over Time: Changes in the Total Score of ER89-R *Note.* ER-89-R = Ego-Resiliency Scale-Revised; T0 = pre-treatment; T6 = half-treatment; T12 = post-treatment; FU = follow-up. Higher scores indicate higher ego-resiliency.

**Figure 6:**
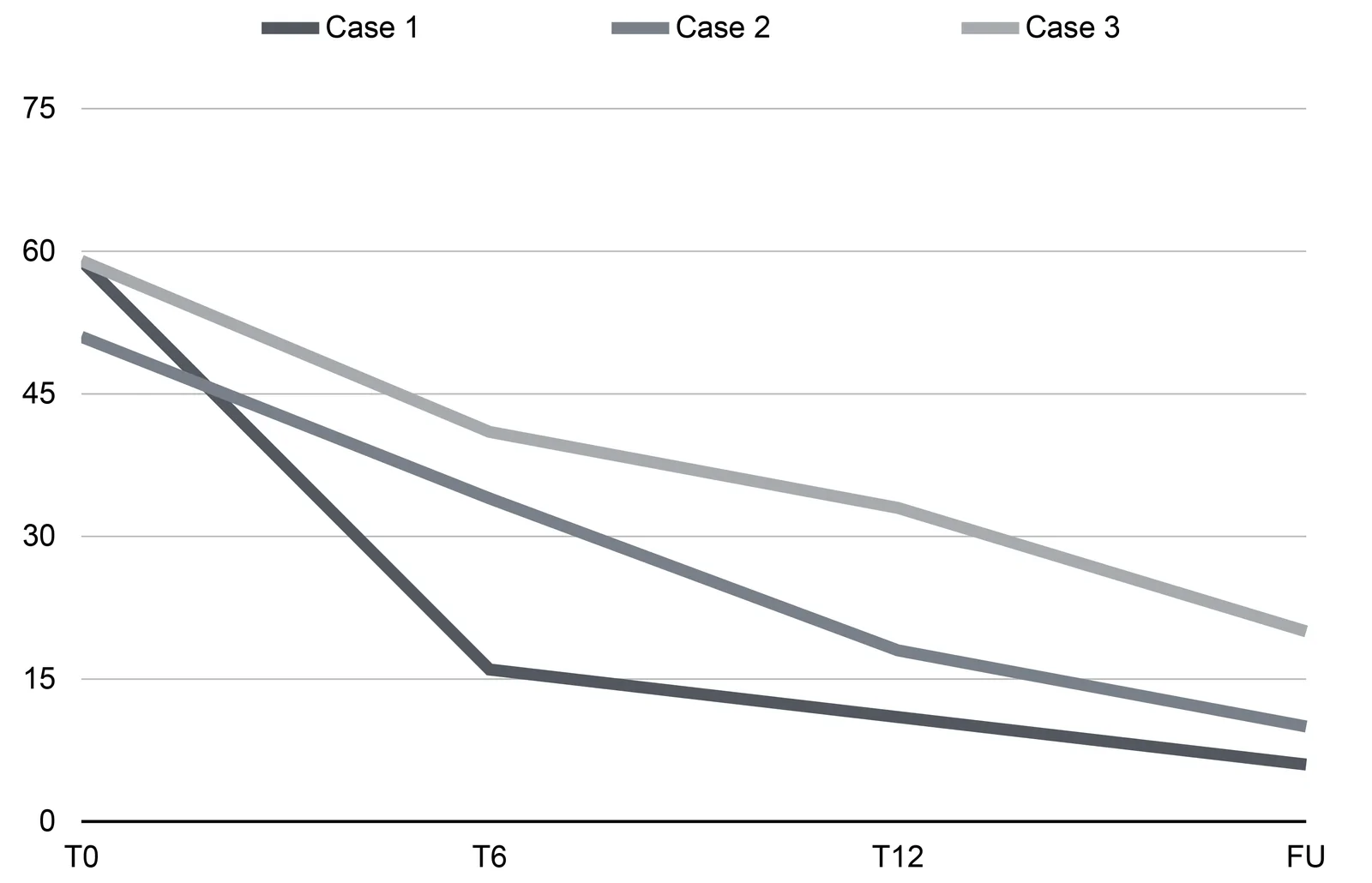
Trauma Symptoms Over Time: Changes in the Total Score of ITQ *Note.* ITQ = International Trauma Questionnaire; T0 = pre-treatment; T6 = half-treatment; T12 = post-treatment; FU = follow-up. Higher scores indicate higher trauma-related symptoms.

**Figure 7:**
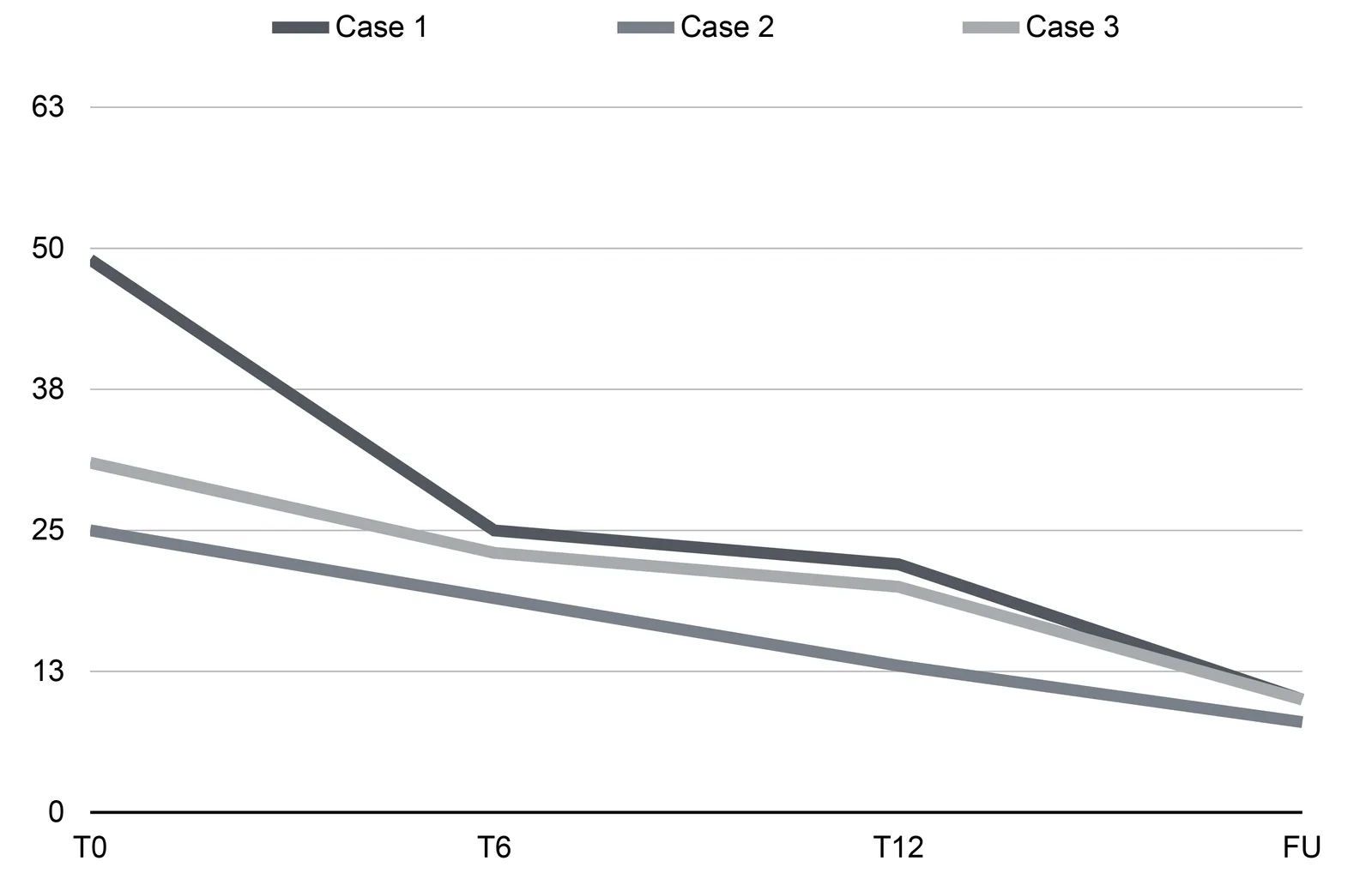
Depression, Anxiety, and Somatic Symptoms Over Time: Changes in the Total Score of PHQ *Note.* PHQ = Patient Health Questionnaire; T0 = pre-treatment; T6 = half-treatment; T12 = post-treatment; FU = follow-up. Higher scores indicate higher depression, anxiety, and somatic symptom severity.

Figures 1, 2, 3, 4, 5, 6, 7 illustrate the longitudinal trajectories of the main outcome measures from baseline to follow-up. Overall, visual inspection showed a consistent reduction in PADS-S and PADS-T scores across the three cases, with particularly marked improvements between baseline and post-treatment that were generally maintained or further improved at follow-up. A similar pattern emerged for ITQ and PHQ scores, which decreased over time in all participants. In parallel, SCS, PGWB, and ER-89-R scores increased across the intervention and follow-up phases. Although the timing and magnitude of change varied across cases and outcome domains, the overall pattern indicated improvement across both symptom-related and resilience-related outcomes. These trajectories are presented descriptively and should be interpreted in relation to the individual case patterns.

## Discussion

To our knowledge, this is the first study to apply the online imagery rescripting (ImRs) to women with pathological affective dependence (PAD) who have survived intimate partner violence (IPV). Across cases, ImRs-PAD was associated with consistent reductions in PAD and other negative symptomatology (complex post-traumatic stress, depression, anxiety), alongside parallel improvements in resilience, self-compassion, and well-being from baseline to follow-up. Taken together, these findings provide preliminary support for the potential efficacy of ImRs in this population. Moreover, they offer initial indications regarding possible mechanisms of change in ImRs (see also Visco-Comandini et al., 2025). Specifically, our results tentatively suggest that addressing unmet relational needs in imagination may contribute to reductions in PAD and related psychological distress, a condition theorized to originate from the chronic frustration of core needs within both the family of origin and abusive partner relationships (Pugliese, Uvelli, et al., 2025).

Evidence from the present study suggests that early maladaptive schemas, as assessed by the YSQ (Young & Brown, 2005), may play a role in shaping individual responses to the intervention (Uvelli et al., 2025). Participants with a predominant self-sacrifice schema appeared to show slower improvement. In contrast, those with more trauma-related schemas (e.g., abandonment, vulnerability) showed earlier reductions in distress following imagery rescripting. Participants with prominent shame/defectiveness schemas showed an intermediate pattern of change, suggesting that different schema profiles may affect both the speed and course of treatment response. Importantly, despite these differences, all participants eventually showed disengagement from abusive relationships. Although these findings are preliminary and based on a small sample, they highlight the potential role of schema-level processes in explaining differences in treatment outcomes.

### Feasibility of Online Delivery

The online delivery of ImRs-PAD appeared acceptable and manageable for all participants. No major technical difficulties, safety concerns, or treatment interruptions were reported during the intervention. The online format may have facilitated engagement by reducing practical and relational barriers to care (i.e., mobility constraints, partner surveillance, and privacy concerns), common among IPV survivors (van Gelder et al., 2023). Although these observations are preliminary and based on a small number of cases, they suggest that online delivery of ImRs-PAD may represent a viable option for trauma-informed care in this population.

### Timing of Therapeutic Change in ImRs-PAD

Improvements in primary and secondary outcomes did not emerge uniformly across participants. This variability may be explained by a combination of relational factors (e.g., type of violence experienced: psychological and/or physical) and contextual factors (e.g., time since separation, ongoing contact with the abusive partner).

#### Relational Risk Factors: The Type of Violence

Differences in the type of violence (physical and/or psychological) experienced may influence the pace of therapeutic change (Jordan et al., 2010). Case 1 primarily reported psychological violence, such as devaluation, control, and verbal abuse, with no indication of severe physical aggression. This pattern may explain her relatively early improvements in PAD, depression, and trauma-related symptoms, as her therapeutic work was not compounded by the more pervasive bodily threat associated with physical violence. Also, Case 3 presented a history of severe psychological and verbal violence, but her therapeutic trajectory was additionally shaped by repeated re-entries into the abusive relationship. These returns prolonged distress and slowed down the stabilization process. However, once the imagery worked effectively, addressing both her fear of abandonment and need for safety, her progress became more consistent and sustainable. By contrast, Case 2 had experienced both psychological and physical violence, with recurrent episodes of intimidation and physical harm. This dual exposure may contribute to her slower therapeutic response compared to Case 1 and Case 3, as reflected in the delayed improvements in PAD and trauma-related symptoms. This pattern is consistent with evidence that combined forms of abuse are linked to more severe psychological consequences and greater treatment resistance (Iverson et al., 2011). Together, these findings suggest that the type of violence—psychological versus combined psychological and physical—may contribute to differential timing of therapeutic gains, with more severe and multifaceted abuse associated with slower change.

#### Contextual Risk Factors: The Separation From the Abusive Partner

Two contextual factors may influence treatment outcomes: (1) the time since the separation from the abusive partner occurred and (2) whether there is an ongoing or intermittent contact versus no current contact with the (ex) partner. Shorter time since last contact and ongoing or intermittent contact are typically linked to less favorable outcomes, including a higher risk of returning to the abusive relationship—a dynamic associated with escalating violence and even femicide (McFarlane et al., 2004). Similarly, Iverson et al. (2011) found that women with current partner contact were less likely to initiate PTSD treatment and showed poorer outcomes compared to those with past but not ongoing abuse. These findings suggest that continued exposure to IPV may undermine both treatment engagement and effectiveness. Our findings are consistent with this evidence. Case 1, who had fully disengaged from the abusive partner for several years, demonstrated the fastest and most stable improvements across outcomes. Case 2, separated for about a year but maintaining intermittent contact, showed the slower progression, with gains consolidating later in treatment. Case 3, who was separated more recently and continued to have active contact with her abusive partner—including repeated returns to the relationship—displayed a mixed trajectory, with delayed stabilization of outcomes. These patterns indicate that the degree of separation from the abusive partner may be another factor that critically shapes the timing and stability of therapeutic change. Nevertheless, despite these contextual challenges, all three cases benefited from ImRs-PAD. This suggests that while achieving a no-contact condition maximizes treatment effectiveness, the intervention can still yield meaningful improvements even when ongoing or intermittent contact with the abusive partner persists.

### Clinical Implications

This study provides preliminary clinical indications that ImRs-PAD may be adapted to address IPV-related difficulties in survivors presenting with PAD. By targeting unmet relational needs for love, safety, and dignity, ImRs offers a therapeutic approach that goes beyond symptom reduction, aiming to modify dysfunctional relational schemas that maintain dependence on abusive partners. Clinically, ImRs-PAD may support disengagement from violent relationships and potentially reducing the likelihood of returning to abusive partners—a situation associated with adverse outcomes, including heightened risk of femicide (McFarlane et al., 2004). These implications must be interpreted cautiously, given the preliminary nature of the evidence, and clinical application should proceed with care until findings are replicated in larger, controlled studies.

### Limitations and Future Directions

This study has several limitations. First, it relied on a multiple-case series design, which limits the ability to draw causal inferences and generalize findings to broader populations. Second, the sample was relatively small and homogeneous, limiting applicability across different cultural, relational, or clinical contexts. Third, outcomes were primarily based on self-report measures, which may be subject to bias. Finally, online delivery may also present specific limitations in this population. For IPV survivors who are still living with a controlling or abusive partner, the home environment may not constitute a safe therapeutic space. Sessions may be subject to monitoring, interruption, or recording. These conditions may reduce the survivor’s perceived safety and interfere with therapeutic openness. For this reason, when delivering the intervention online, particular attention should be paid to safety conditions. Therapists should ensure that patients have access to a private and secure space for the session and can interrupt it at any time if they feel at risk. Moreover, given the potentially reparative role of the therapeutic relationship in trauma-related relational difficulties, the absence of physical co-presence may affect engagement and treatment outcomes. In this regard, the possibility of subsequent in-person treatment should be considered, when feasible, to further support the therapeutic process and enhance the reparative potential of the therapeutic relationship. Future research should directly compare online and in-person delivery modalities.

Despite these limitations, the findings are encouraging and highlight new directions for future research. Controlled studies with larger and more diverse samples are needed to further evaluate the effectiveness of ImRs-PAD and to identify predictors of early versus late treatment response. Once preliminary efficacy has been established, future studies may explore relational, contextual, and personality factors as potential moderators. For example, ongoing contact with the abusive partner, the type of violence (physical, sexual, psychological, or verbal), and/or specific schema may critically shape the pace and stability of improvements. Furthermore, dismantling studies could help clarify the relative contributions of early-life rescripting versus interventions targeting current and future partner-related dynamics. Longitudinal follow-up at three, six, and twelve months will be essential to determine whether the benefits of ImRs-PAD are sustained over time and translate into safer relational choices. In addition, incorporating behavioral outcomes—such as the intention to return to the abusive partner—and biological indicators, including cortisol, micro-RNAs, and inflammatory markers, could provide deeper insight into how ImRs-PAD supports survivors in breaking free from PAD and IPV. Finally, dedicated studies are needed to clarify the mechanisms of change in ImRs-PAD, showing how addressing unmet needs during rescripting translates into therapeutic gains.

### Conclusion

This multiple case series provides preliminary support for the effectiveness of ImRs-PAD, a specific three-stage ImRs protocol grounded in PAD theory (Pugliese et al., 2023). According to this theory, PAD originates from the frustration of core needs—being loved, feeling safe, and experiencing self-worth—within childhood caregiving and adult intimate relationships. PAD may represent a relevant psychological risk pathway for IPV (Pugliese, Papa, et al., 2025; Pugliese, Uvelli, et al., 2025). After addressing these memories in guided imaginations and meeting the unmet relational needs during rescripting, we observed reductions in PAD symptoms, along with improvements in well-being, resilience, and self-compassion, as well as decreases in depression, anxiety, trauma-related, and somatic symptoms. These findings broaden the use of ImRs, showing its relevance for PAD and IPV survivors. They also contribute to a better understanding of the mechanisms of change in ImRs.

## Supplementary Materials

The appendix associated with this article includes the following contents (for access, see Pugliese et al., 2026S):

- Appendix A. Detailed description of the ImRs-PAD sessions. The appendix provides an overview of the structure, aims, and clinical focus of each session of the ImRs-PAD intervention.

PuglieseE.
UvelliA.
van EmmerikA. A. P.
ArntzA.
 (2026S). Supplementary materials to "Online application of imagery rescripting for pathological affective dependence (ImRs-PAD) in survivors of intimate partner violence: Results from a multiple case series"
[Appendix]. PsychOpen. 10.23668/psycharchives.22357


## Data Availability

The data that support the findings of this study are available from the corresponding author upon reasonable request.

## Preregistration

This manuscript reports a preliminary evaluation of ImRs-PAD conducted within the framework of a broader preregistered clinical study. The full study protocol is registered at ClinicalTrials.gov under the identifier NCT06670326. The present article reports the preliminary findings from the intervention protocol before completion of the full study. Therefore, the findings should be interpreted as preliminary and exploratory in relation to the broader preregistered trial.

## Reporting Guidelines

The manuscript was prepared in accordance with the CARE guidelines for case reports and case series.

## Social Media Accounts

*Erica Pugliese:* LinkedIn
